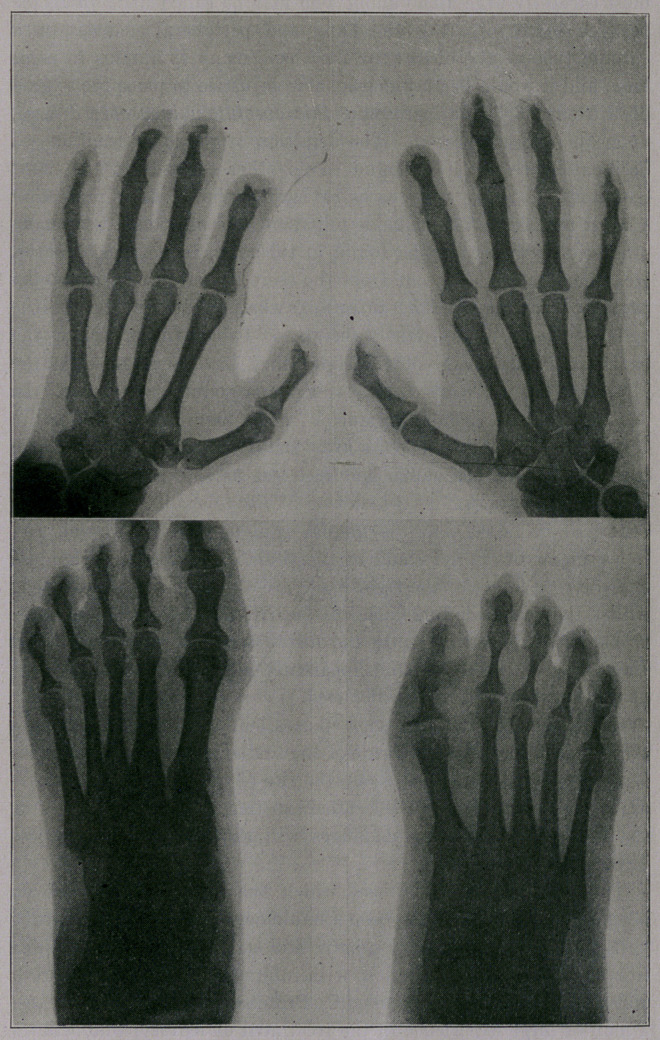# Report of a Case of Early Bone Lesion in Leprosy, with Remarks and Radiograms

**Published:** 1911-07

**Authors:** R. H. L. Bibb

**Affiliations:** Saltillo, Mexico


### TEXAS MEDICAL JOURNAL.

Established July, 1885

F. E. DANIEL, M. D., - - - - - - Editor, Publisher and Proprieto

Published Monthly.—Subscription $1.00 a Year.

VOL. XXVII. AUSTIN, JULY, 1911. No. 1.

The publisher is not responsible for the views of the contributors.

### Original Articles.

For Texas Medical Journal.

### Report of a Case of Early Bone Lesion in Leprosy, with Remarks and Radiograms.

BY R. H. L. BIBB, M. D., SALTILLO, MEXICO.

Volumes have been written on leprosy, to which literature the writer has added, perhaps, more than one knowing no more about the disease than he does, should have added. Be that as it may, leprosy has been of deepest interest, to medical men and to laymen, ever since Moses taught the priests how to cure the disease—a knowledge which, sad to relate, has been lost in the ages which have come and which have gone, *for there is no living man that knows of any method by which a single case of leprosy can be cured.*

This assertion, as will be noted by some, is at variance with opinions which I formerly held, and which I have voiced on other occasions; but being based upon a better understanding of the malady, and a more extended experience in its management, coupled with their consonance with the opinions of others whose large and varied experiences with leprosy entitle them to be heard, is, I am quite sure, entirely correct. Hence I have no hesitancy in repeating that the disease should be classed among the incurable.

Barring the nasal bones, leprosy as a rule attacks the osseous structures only when the disease is very far advanced. It is for this reason that I beg to call attention to the following case, in which the bones have been attacked quite early, and at a distance from the usual seat of bone involvement in leprosy.

Senorita ———, aged 19 years, consulted me three years ago for a numbness in the extremities of the fingers and toes. This numbness being most marked in the "tips" of these members. So pronounced was it that she would often burn, prick and otherwise injure them without any knowledge of the fact until some accident would call her attention to the lesions, some of which were of considerable importance. On one occasion she actually sewed her left index finger to the garment she was making. This loss of sensation—growing gradually less toward the trunk—extended nearly to the elbows and knees. Pressure along the course of the nerve trunks caused rather an agreeable sensation of "tingling" as if the member were "asleep." She noted the loss of sensation about six months before consulting me. It had very gradually grown worse.

Several months after her first consultation, she claimed to have observed that some of her fingers were becoming shorter and stubbier; that the anesthesia was more pronounced; that some of her toes were shorter and that the nails were curving downward and assuming the shape of an animal's claws. She also called my attention to what appeared to be a perforating ulcer on the plantar surface of the joint of the basilar, with the middle phalanx of the left great toe. The ulcer was painless. She denied having suffered pain, either in the ulcer or in either extremity at any time, and insisted that the sensation had never been other than a pleasant, tingling numbness. This absence of pain, especially along the course of the large nerve trunks, is exceedingly rare in leprosy, and I am at a complete loss to explain it in this case.

I attended the young lady's mother when the patient was born. I have known the family for a quarter of a century, every branch of it, and with one exception the family history is good. Her father, mother, brothers, sisters and grandparents are and were strong, healthy, robust people, excepting her maternal grandmother, who died several years before the patient was born, at the age of sixty, of pneumonia—who showed unmistakable symptoms of tubercular leprosy—Hansen's bacillae were found in enormous numbers in a tubercle taken from the lobe of her right ear. So there can be no mistake as to the diagnosis in that case.

An examination of the pus from the plantar ulcer showed an abundance of acid-fast bacillae which, with the carbol-fuchsin stain, met the requirements of the lepra bacillus, and a diagnosis was made accordingly. I was fully persuaded, however, that the case was one of leprosy; but in order to make assurance doubly sure, and in obedience to a rule which I have followed for a quarter of a century, viz., never to make a positive diagnosis of leprosy until the bacillus is found which is reputed to be the cause of the disease, but which has never been proven to be, unless the symptoms are so plain that "he who runs may read," a rule which I most earnestly urge all to adopt who may be called upon to decide between leprosy on the one hand and peripheral neuritis of a chronic type on the other; for they have many symptoms in common, and it would be an unpardonable injustice to place the stigma of this terrible, this loathsome, *this absolutely incurable disease,* upon an innocent person. The detection of the lepra bacillus—so designated because it is found in no other disease—in the nasal secretions of many early cases of leprosy should be a protection against mistakes, alike valuable to patient and physician. In many others the bacillus may be found in tubercles, in nodules, fissures, etc., should they be absent from the secretions from the nose—the portal of entrance of the infection, whatever it may be, in the belief of many competent observers. I have never found the bacillus in the blood—others claim they have—except when the blood was taken directly from a tubercle, a nodule or a fissure. In one case I found them in scrapings from a fissure of the lower lip.

Six months ago, three years after the patient first consulted me, the condition of her hands and feet was as shown in the accompanying radiograms.

It will be noted that the ungual phalanges of the thumb and index finger of the left, and of the thumb, the index, the middle and the ring finger of the right hand, are almost entirely destroyed; whilst the corresponding bones of both little fingers show considerable atrophy, leaving only the distal bones of the middle and ring finger of the right hand apparently normal. The nails of all of the fingers and toes are distorted.

Radiograms of the feet show that all of the ungual phalanges are diseased, and that the heads of the basilar and middle phalanges of the great toe of the left foot—the site of the perforating ulcer—are quite destroyed. Indeed, I believe that a careful scrutiny of the heads of all of the phalanges will show them more or less implicated.

The treatment in this case, which has been absolutely of no benefit that I can see, has been Chaulmoogra oil internally, externally and locally, with tonics, baths and such hygiene as is possible to be had on a Mexican ranch, with intervals of chlorate of potash and strychnine, as recommended by Professor Dyer of New Orleans, a man of large experience in the treatment of leprosy, who believes he has cured cases of it.

The consensus of opinion concerning the proper disposition of leper patients is that each individual should be completely isolated.

I may add that the combined experience of the world teaches us that isolation is the only successful way to deal with it. No method of treatment can be relied upon to do more than mitigate the disease, and mitigation offers no protection to the public. There are a half a hundred or more of unrestrained lepers in Texas now; and if proper disposition is not made of them ere it be too late, future generations may anathema maranatha those whose duty it is, if such there are, should they fail to afford them that protection which the history of leprosy teaches to be isolation, only isolation, in the strictest acceptation of the term. They should all be segregated and isolated during life, and after death their bodies should be cremated, together with all of their personal belongings.

Bouchard, in his classical work on "Infectious Disease," declares that "whenever and wherever man has wished to do so, he has been able to liberate himself from the ravages of leprosy, and that it may be entirely extinguished by proper isolation." Hansen says: "There can hardly be any doubt that segregation is the only right way, at least, after our experience here in Norway for stamping out leprosy, and that there are grounds for hope for the diminution and even the ultimate extermination of leprosy in most parts of the world by proper isolation and segregation." Roose believes that the rigid system of isolation in vogue in Norway will, in a few years, work complete extinction of the disease. Wheeler thinks leprosy can be eradicated by separating the sexes in asylums. Goldschmidt, that complete isolation of all lepers and their families is the only reliable measure in order to quickly and totally eradicate the contagion and ultimately making this loathsome disease completely disappear from the face of the earth. Hellat, member and founder of the chief committee for stamping out leprosy in the Baltic providences of Russia, writes that the theory of infection points out the way by which we may arrive at the annihilation of leprosy, for so long, he writes, as it must be looked upon as *incurable,* isolation alone can lead to the goal. Münch, a contagionist, considers the only means of eradicating the disease is to isolate. He asserts that isolation in a given locality stamps out leprosy, and cites many instances in southern Russia in substantiation of his assertions.

Excerpts of like import from the writings of other equally eminent leprologists might be added, almost *ad libitum;* but those which I have cited are enough to indicate the trend of thought on this important phase of the leper subject.

In conclusion, I believe that we are warranted in saying:

1. That leprosy is a specific disease, caused, most probably, by the lepra bacilli.

2. That leprosy is influenced by race, climate, soil, food, etc., only in so far as these conditions tend to enervation on the one hand, or to physical well being on the other.

3. That experiments have not demonstrated leprosy to be inoculable on man or beast.

4. That leprosy is hereditary in the same sense that is its congener, tuberculosis.

5. That leprosy is contagious, infectious and communicable, under conditions not yet understood.

6. That whilst mitigable in some cases, leprosy must be regarded as an incurable disease.

7. *That by proper isolation and segregation, leprosy may be completely eradicated from the list of human ills.*

## Figures and Tables

**Figure f1:**